# Detection of ‘best’ positive end-expiratory pressure derived from electrical impedance tomography parameters during a decremental positive end-expiratory pressure trial

**DOI:** 10.1186/cc13866

**Published:** 2014-05-10

**Authors:** Paul Blankman, Djo Hasan, Groot Jebbink Erik, Diederik Gommers

**Affiliations:** 1 Department of Intensive Care Adults, Erasmus MC Rotterdam, Room H623, ‘s Gravendijkwal 230, Rotterdam 3015 CE, The Netherlands; 2 Department of Intensive Care, Maasstad Hospital, Maasstadweg 21, Rotterdam 3079 DZ, The Netherlands; 3 Institute of Technical Medicine, University of Twente, Drienerlolaan 5, Enschede 7522 NB, The Netherlands

## Abstract

**Introduction:**

This study compares different parameters derived from electrical impedance tomography (EIT) data to define ‘best’ positive end-expiratory pressure (PEEP) during a decremental PEEP trial in mechanically-ventilated patients. ‘Best’ PEEP is regarded as minimal lung collapse and overdistention in order to prevent ventilator-induced lung injury.

**Methods:**

A decremental PEEP trial (from 15 to 0 cm H_2_O PEEP in 4 steps) was performed in 12 post-cardiac surgery patients on the ICU. At each PEEP step, EIT measurements were performed and from this data the following were calculated: tidal impedance variation (TIV), regional compliance, ventilation surface area (VSA), center of ventilation (COV), regional ventilation delay (RVD index), global inhomogeneity (GI index), and intratidal gas distribution. From the latter parameter we developed the ITV index as a new homogeneity parameter. The EIT parameters were compared with dynamic compliance and the PaO_2_/FiO_2_ ratio.

**Results:**

Dynamic compliance and the PaO_2_/FiO_2_ ratio had the highest value at 10 and 15 cm H_2_O PEEP, respectively. TIV, regional compliance and VSA had a maximum value at 5 cm H_2_O PEEP for the non-dependent lung region and a maximal value at 15 cm H_2_O PEEP for the dependent lung region. GI index showed the lowest value at 10 cm H_2_O PEEP, whereas for COV and the RVD index this was at 15 cm H_2_O PEEP. The intratidal gas distribution showed an equal contribution of both lung regions at a specific PEEP level in each patient.

**Conclusion:**

In post-cardiac surgery patients, the ITV index was comparable with dynamic compliance to indicate ‘best’ PEEP. The ITV index can visualize the PEEP level at which ventilation of the non-dependent region is diminished, indicating overdistention. Additional studies should test whether application of this specific PEEP level leads to better outcome and also confirm these results in patients with acute respiratory distress syndrome.

## Introduction

Mechanical ventilation acts as a stress raiser to lung tissue adjacent to collapsed tissue in the dependent lung and the risk of alveolar hyperinflation in the non-dependent lung [1]. An alveolar recruitment maneuver and the use of positive end-expiratory pressure (PEEP) are applied to open up and to keep open the atelectatic lung tissue in an attempt to minimize this stress and over-distention.

The original definition of the best PEEP as proposed by Suter *et al*. is the PEEP level with the best compromise between lung aeration and circulatory depression [2]; circulatory depression is caused by compression of capillaries due to hyperinflation. Tusman *et al*. performed an incremental and decremental PEEP trial in eight volume-controlled ventilated surfactant-depleted pigs; they showed that calculation of dead space detected early signs of lung collapse, which correlated well with findings on computed tomography (CT) [3]. In a second study, these authors reported that continuous compliance monitoring could identify the onset of alveolar collapse as confirmed by CT, as well as changes in partial arterial oxygen pressure (PaO_2_) values [4]. Another experimental study, using an oleic acid-induced acute lung injury (ALI) model, showed that minimizing elastance of the respiratory system could be used to titrate PEEP settings [5]. Maisch *et al*. performed an incremental and decremental PEEP trial in 20 anesthetized patients undergoing elective surgery and confirmed that dynamic compliance and dead-space calculations were able to detect alveolar collapse and/or over-distention, whereas functional residual capacity (FRC) and PaO_2_ changes were less sensitive [6]. From these studies it has been concluded that dynamic compliance and dead-space calculations are the most reliable global parameters to define the best PEEP at the bedside.

CT is regarded as the gold standard to assess the effect of a recruitment maneuver and the applied PEEP on aeration of the lung [7,8]. However, the obvious drawbacks of repeated CT scans (that is, transfer of the mechanically ventilated patient and excessive radiation exposure) reduce the application of CT as a tool for assessment of PEEP settings. On the other hand, electrical impedance tomography (EIT) is a real-time bedside monitoring tool, which has proven to correlate well with CT for assessment of changes in gas volume and tidal volume [9-11].

Several EIT parameters have been developed to collect more data on ventilation distribution in order to optimize ventilator settings [12-15]. The present study examines whether one specific EIT parameter is able to describe the optimal PEEP level at the bedside; for this, we defined the best PEEP as the PEEP level with minimal lung collapse and minimal over-distention.

## Materials and methods

### Study population

Included in this study were 12 mechanically ventilated post cardiac surgery patients admitted to the cardiothoracic intensive care unit. Data for the present study were used in an earlier study that analyzed the effect of a decremental PEEP trial on ventilation distribution with EIT measured at two different thoracic levels [16]. Informed consent was obtained from the patient or a legal representative. Using data from this latter study, we re-analyzed data of the EIT measurements made just above the diaphragm only, as this part of the lung is at most risk for formation of atelectasis in mechanically ventilated patients in the supine position. The Medical Ethical Committee Rotterdam approved the entire study protocol.

### Study protocol and measurements

A 16-electrode silicon belt (EIT evaluation kit 2, Dräger, Lübeck, Germany) was placed around the patient’s thoracic cage between the 6th and 7th intercostal spaces [16]. Patients were ventilated with pressure-controlled ventilation (PCV) (Engström Carestation, GE Healthcare, Madison, WI, USA) and, throughout the entire study period, the inspiratory pressure above PEEP, the Inspiration/expiration (I/E) ratio, frequency and inspired oxygen fraction (FiO_2_) remained unchanged.

In this study we performed a recruitment maneuver in which mechanical ventilation was continued with a pressure amplitude of 20 cm H_2_O while PEEP was rapidly increased from 5 to 20 cm H_2_O in incremental steps of 5 cm H_2_O: thus, a peak pressure of 40 cm H_2_O for a 40-s period, as long as blood pressure remained stable. Thereafter, PEEP was decreased to 15 cm H_2_O and the pressure amplitude was decreased from 20 to 10 cm H_2_O. A PEEP level of 15 cm H_2_O was applied for 15 minutes to achieve a steady state. Thereafter, a decremental PEEP trial was performed from 15 to 0 cm H_2_O PEEP in steps of 5 cm H_2_O. Each PEEP level was applied for 10 to 20 minutes (depending on hemodynamic stability and blood gas analyses). At the end of each PEEP step, EIT, PaO_2_/FiO_2_ ratio and dynamic compliance (tidal volume divided by pressure above PEEP) were calculated.

### EIT data analysis

**Figure 1:**
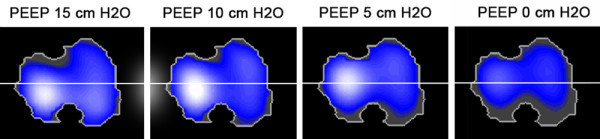
**Example of electrical impedance tomography (EIT) image reconstruction.** Shown is the distribution of impedance to the dependent and non-dependent lung regions of one representative patient. The lighter the color, the higher the impedance and the more aerated the lung region. The surface area used in all calculations at every positive end-expiratory pressure (PEEP) step is equal to the largest surface area. The EIT image is divided into two equal regions of interest represented by the white line; all EIT modalities are calculated using this setup. Decreasing the PEEP value resulted in a decrease in aeration of the lungs, especially in the dependent region.

EIT data were recorded with a sample rate of 20 Hz and were analyzed using dedicated software (EITdiag, Dräger Medical, Lübeck, Germany). For each PEEP step a stable phase with 10 to 20 consecutive breaths was selected. The EIT signals of these breaths are filtered using a low-pass filter set on 40 beats per minute to minimize signals induced by the cardiovascular system. From the filtered signals a ventilation distribution map was created for each PEEP step (Figure 1). The surface of the distribution maps was standardized, using the largest EIT image acquired during the PEEP trial for each patient. The EIT signals in the distribution maps are used to calculate the tidal impedance variation (TIV), ventilation surface area (VSA), center of gravity (COG), and the global inhomogeneity (GI index). In order to reliably calculate the intratidal gas distribution, all filtered signals were resampled at 40 Hz to divide the inspiratory part of the TIV curve more accurately into 8-iso volume steps. To calculate the different parameters, the defined surface area was divided into two equal regions of interest, that is, the dependent and non-dependent regions (Figure 1).

### Calculated EIT parameters

EIT measures changes in electrical impedance between electrode pairs. After adequate filtering of EIT signals, the measured impedance changes represent the inspiration and expiration by means of TIV (formula 1), which correlates well with tidal volume [9,11,17,18].

(1) $$\text{TIV}=\text{Impedanc}e_{\text{max}}-\text{Impedanc}e_{\text{min}}$$

(TIV = Tidal Impedance Variation)

The second EIT parameter to be calculated is regional compliance [12]. Calculation of regional compliance is similar to that of dynamic compliance; however, for dynamic compliance tidal volume is divided by pressure amplitude whereas for regional compliance TIV is divided by pressure amplitude. As we did not connect the EIT device to the ventilator, we were unable to calculate regional compliance using EITdiag software. Therefore, we divided the TIV into dependent and non-dependent lung regions by the EITdiag-generated ventilation distribution map, based on the pressure above PEEP (formula 2). A decrease in regional compliance with increases in PEEP indicates that the lung is hyperinflated, whereas a decrease in regional compliance with decreases in PEEP indicates alveolar collapse.

(2) $${\text{Compliance}}_{\text{region}}=\frac{{\text{TIV}}_{\text{region}}}{\text{Pressure}\;\text{above}\;\text{PEEP}}$$

(TIV = Tidal Impedance Variation)

Using EITdiag software, we calculated the VSA. For this, the number of pixels with an EIT signal in the generated ventilation distribution map was counted for both lung regions. During the recruitment maneuver, the number of ventilated pixels per region is divided by the total number of ventilated pixels in the ventilation distribution map, assuming that all recruitable lung tissue was open at the end of recruitment. In this way, regional ventilation distribution is expressed as a percentage of the maximum ventilated pixels at the end of recruitment. After increasing PEEP levels, higher VSA values indicate alveolar recruitment whereas after decreasing PEEP levels higher VSA values indicate that the lungs were hyperinflated during the previous PEEP level.

In the present study, calculation of the regional ventilation delay (RVD) index describes the percentage of time needed to reach a threshold of 40% of the regional impedance changes, as compared with the total inspiratory time (formula 3) [15,19]. This calculation was not performed using the EITdiag software. Large differences in RVD between both lung regions indicate that the lungs are inhomogeneously ventilated.

(3) $$\mathrm{RV}D_{i}=\frac{\Delta t_{i}^{40\%}}{t_{\max}-t_{\min}}\times 100\%$$

(RVD = Regional Ventilation Delay index; i = region; Δ = delta)

The intratidal gas distribution was analyzed according to Löwhagen *et al*. [14] (formula 4), which is integrated in the EITdiag software. To calculate the intratidal gas distribution the inspiratory part of the global TIV curve is divided into eight iso-volume parts. Thereafter, the eight corresponding time points are translated to the regional TIV curves. Using this technique, we calculated the percentile contribution of the dependent and non-dependent regions to the inspiration. Thereafter, we developed the intratidal gas distribution index (ITV index) to evaluate whether the lung is homogeneously ventilated. The ITV index is calculated by dividing the ITV of the non-dependent lung region by the ITV of the dependent region (formula 5). An ITV index of 1 indicates an equal distribution of ventilation to the dependent and non-dependent lung regions.

(4) $$\text{Fractional}\;\text{regional}\;{\mathrm{ITV}}_{1-8}=\frac{{\text{ITV}}_{1-8}{\mathrm{TIV}}_{\mathrm{ROI}}}{{\mathrm{ITV}}_{1-8}{\mathrm{TIV}}_{\text{Global}}}$$

(5) $$\text{ITV}-\text{index}=\frac{\sum_{r1,E}\mathrm{ITV}\;\text{non}-\text{dependent}}{\sum_{r1,E}\text{ITV}\;\text{dependent}}$$

(ITV = Intratidal Gas Distribution; TIV = Tidal Impedance Variation; ROI = Region of Interest; t = iso-volume part)

The COV reflects the distribution of tidal ventilation in the ventral-to-dorsal direction [13] (formula 6). Therefore, the TIV of the dependent region is divided by the total TIV of the EIT image. When most of the tidal ventilation distributes to the dependent lung region this results in a small COV value. We constructed a plot of the centers of ventilation to visualize the shifts in regional lung ventilation in the anterior-to-posterior direction during the PEEP trial.

(6) $$\mathrm{COV}=\frac{{\mathrm{TIV}}_{\text{dorsal}}}{{\mathrm{TIV}}_{\text{total}}}$$

(COV = Center of Ventilation; TIV = Tidal Impedance Variation)

The GI index, developed by Zhao *et al*., quantifies ventilation distribution in the lungs [20,21]. Therefore, the tidal impedance difference per pixel is subtracted by the median tidal impedance difference of the lung. Thereafter, the result of this subtraction is divided by the total impedance changes of all pixels in order to normalize the calculated values (formula 7). Thus, the GI index calculates the variance in impedance per pixel as compared with the total EIT image. The smaller the GI index, the more homogeneous the lung ventilation. This calculation is integrated in the EITdiag software.

(7) $$\mathrm{GI}=\frac{\sum_{x,y\in \mathrm{lung}}|\text{Impedance}\;{\text{difference}}_{\mathrm{xy}}-\text{Median}\;\left(\text{Impedance}\;{\text{difference}}_{\text{lung}}\right)|}{\sum_{x,y\in \text{lung}}\text{Impedance}\;{\text{difference}}_{\mathrm{xy}}}$$

$$\begin{array}{l} \text{GI}\;\text{index}\;\text{formula}:(x\;\text{and}\;y\;\text{describe}\;\text{the}\;\text{location}\;\text{of}\;\text{the}\;\text{pixel}\;\text{on}\\ \;\text{the}\;x\;\text{and}\;y\;\text{axes};\in =\text{element}\;\text{of}\;\text{ventilated}\\ \;\text{part}\;\text{of}\;\text{the}\;\text{EIT}\;\text{image}) \end{array}$$

### Statistical analysis

Statistical analyses were performed using SPSS version 21 (IBM, Chicago, IL, USA). Unless specified otherwise, values are presented as means ± SD. Data were tested for normal distribution and homoscedasticity using the Kolmogorov-Smirnov test and the Brown-Forsythe test. If the data had a normal distribution we applied analysis of variance (ANOVA), otherwise, the independent-samples Kruskal-Wallis test was used. Differences in PaO_2_/FiO_2_ ratio and dynamic compliance between the PEEP steps were analyzed using mixed linear model analyses. Correlation between the PaO_2_/FiO_2_ ratio and ITV index was calculated using the two-tailed Spearman rho test.

All *P*-values <0.05 are considered to be statistically significant.

## Results

**Figure 2:**
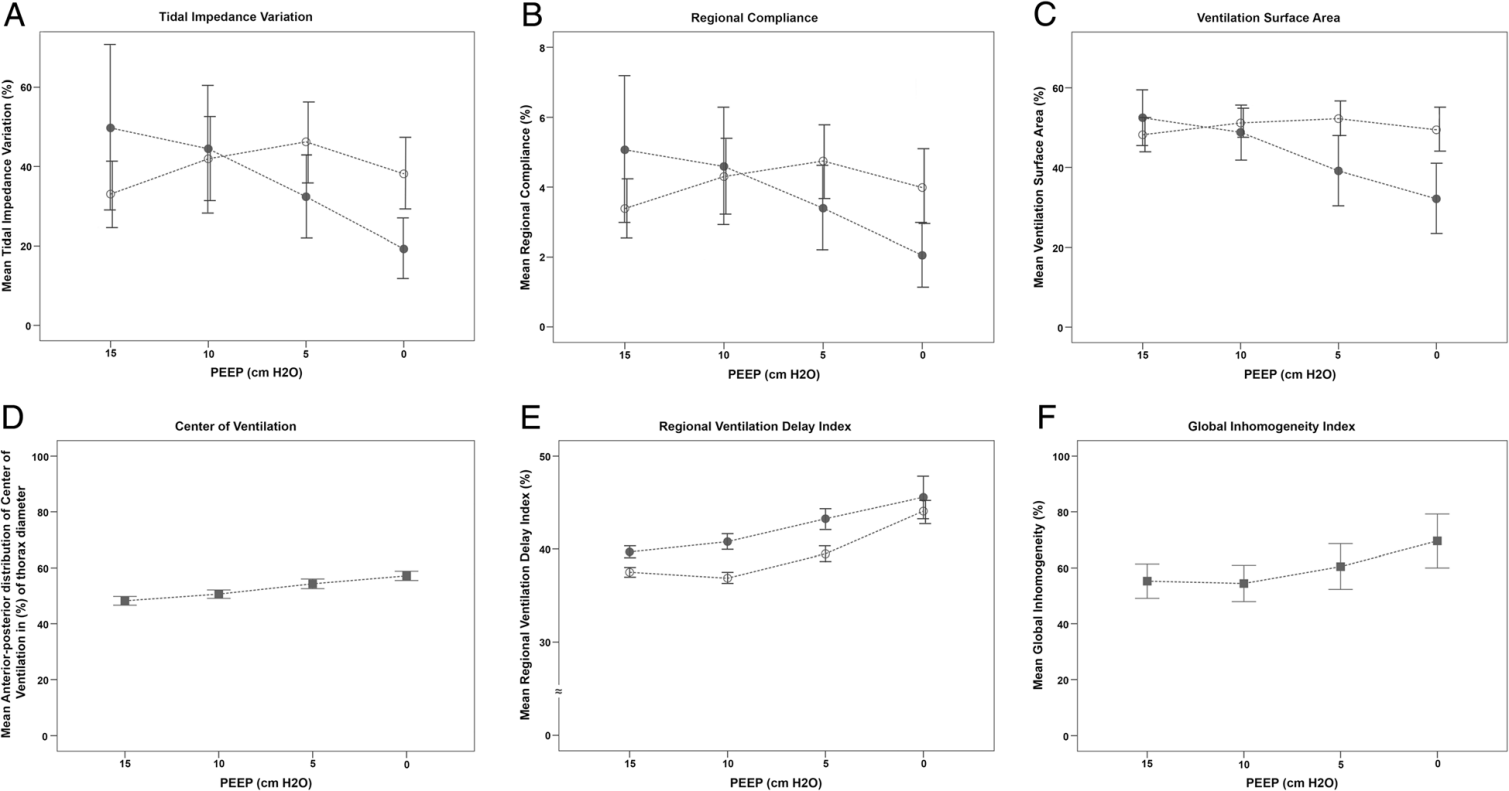
**Different electrical impedance tomography (EIT) modalities calculated for the decremental positive end-expiratory pressure (PEEP) trial.** Effects of different PEEP levels on regional changes in **(A)** tidal impedance variation (TIV); **(B)** regional compliance; **(C)** ventilation surface area (VSA); **(D)** center of ventilation (COV); **(E)** regional ventilation delay (RVD) index; and **(F)** global inhomogeneity (GI) index. Data are presented as means ± 95% CI. Dashed lines represent the interpolation lines; open circles = non-dependent regions; solid circles = dependent regions; solid squares = entire EIT image.

Details of patient characteristics are presented in Table 1. During the entire PEEP trial, patients were ventilated with an inspiratory pressure above PEEP of 10 ± 2 cm H_2_O. A PaO_2_/FiO_2_ ratio ≥350 mmHg was defined as an open lung; in two patients we were unable to open up the lung despite the recruitment maneuver and use of a PEEP level of 15 cm H_2_O. Figure 1 shows the distribution of TIV for one representative patient during the decremental PEEP trial. The effects of decremental PEEP on TIV, regional compliance, VSA, COV, RVD and GI index are presented in Figure 2A-F.

**Table 1:** Baseline characteristics of the study population

**Characteristic**	**Value**
Number of patients	12
Age, years	70 ± 9
Male:female, number	9:3
Weight, kg	79 ± 12
PBW, kg	67 ± 9
Height, m	1.72 ± 0.08
Body mass index	27 ± 4
Respiratory rate, breaths per minute	15 ± 1
PIP, cm H_2_O	25 ± 2
V_Te_, mL	591 ± 120
V_T_/PBW, mL/kg	8.8 ± 1.7

Data are presented as means ± SD, unless stated otherwise. PBW, predicted body weight; PIP, peak inspiratory pressure; V_Te_, expiratory tidal volume.

**Figure 3:**
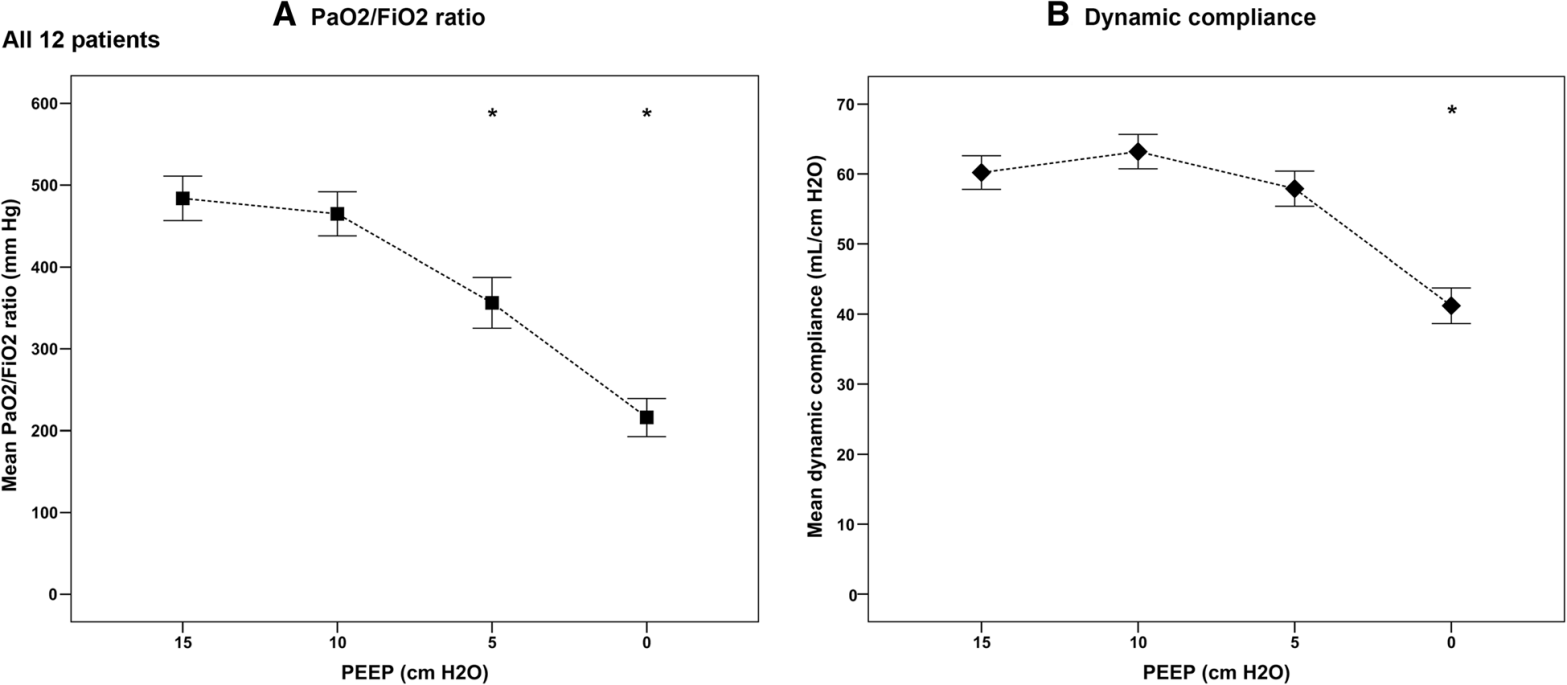
**Changes in partial arterial oxygen pressure (PaO**_**2**_**)/inspired oxygen fraction (FiO**_**2**_**) ratio and dynamic compliance during a decremental positive end-expiratory pressure (PEEP) trial. (A)** The PaO_2_/FiO_2_ ratio (mmHg) and **(B)** dynamic compliance (mL/cm H_2_O) are shown for the entire group. The PaO_2_/FiO_2_ ratio decreased with every PEEP step. The PaO_2_/FiO_2_ ratio showed a significant decrease at 5 and 0 cm H_2_O PEEP compared with 15 cm H_2_O PEEP. Dynamic compliance increased after reducing PEEP from 15 to 10 cm H_2_O. Thereafter, dynamic compliance decreased with each PEEP step. At 0 cm H_2_O PEEP dynamic compliance was significantly reduced compared with 15 cm H_2_O PEEP. Solid squares = PaO_2_/FiO_2_ ratio; solid diamonds = dynamic compliance. *P <0.05.

Figure 3 shows the effects of the decremental PEEP trial on the PaO_2_/FiO_2_ ratio and dynamic compliance for the entire study population. The PaO_2_/FiO_2_ ratio had the highest value at 15 cm H_2_O PEEP but showed a significant decrease after lowering PEEP from 10 to 5 to 0 cm H_2_O compared with 15 cm H_2_O PEEP (Figure 3A). Dynamic compliance had the highest value at 10 cm H_2_O PEEP but showed a significant decrease at a PEEP level of 0 compared with 15 cm H_2_O (Figure 3B).

In the non-dependent lung regions, TIV (Figure 2A), regional compliance (Figure 2B) and VSA (Figure 2C) reached the maximum value at 5 cm H_2_O PEEP. In contrast, in the dependent region TIV, VSA and regional compliance reached a maximum at the highest PEEP level applied and then decreased during the entire PEEP trial. During the decremental PEEP trial, COV increased steadily towards the anterior part of the thorax cavity, indicating loss of TIV in the dependent region (Figure 2D). During the decremental PEEP trial the RVD index increased in both lung regions and the RVD values of the non-dependent region remained significantly lower than those in the dependent region, except at zero end-expiratory pressure (Figure 2E). The GI index had the lowest values at 15 and 10 cm H_2_O PEEP and then increased steadily at lower PEEP levels (Figure 2F), indicating more homogeneous ventilation at higher PEEP levels.

**Figure 4:**
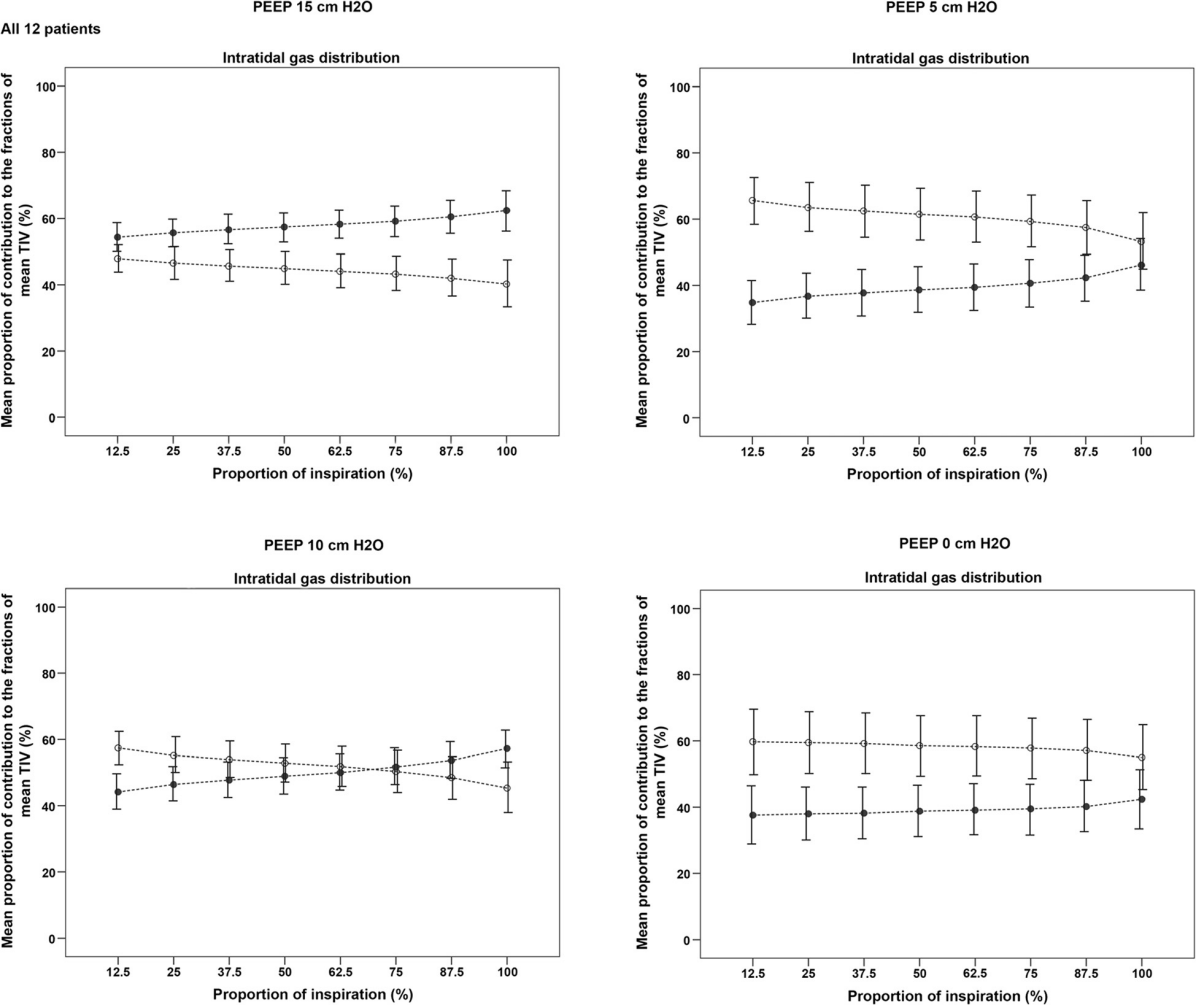
**Intratidal gas distribution at varying positive end-expiratory pressure (PEEP) levels.** Mean intratidal gas distribution in eight iso-volume steps during four PEEP levels during the decremental PEEP trial. Decreasing the PEEP level resulted in a higher overall gas distribution to the non-dependent region and, subsequently, a lower gas distribution to the dependent region. During the course of inspiration, gas distribution to the non-dependent region decreased whereas it increased to the dependent region. At a PEEP level of 10 cm H_2_O, during inspiration the lines representing gas distribution to both regions crossed each other. Dashed lines represent the interpolation lines; open circles = non-dependent region; solid circles = dependent region.

**Figure 5:**
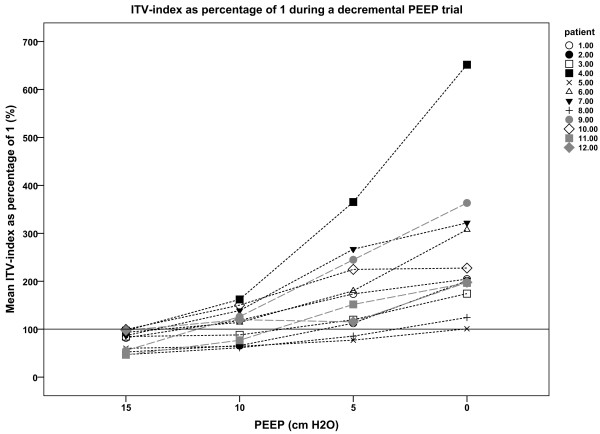
**Intratidal gas distribution (ITV) index at varying positive end-expiratory pressure (PEEP) levels for the individual patients.** Mean ITV index is shown as a percentage of 1. Here, the value 100% indicates that both lung regions are equally ventilated. Values >100% indicate that ventilation is predominantly distributed to the non-dependent region whereas values <100% indicate that the dependent lung region is predominantly at that PEEP level.

Figure 4 presents the results of intratidal gas distribution. At the highest levels of PEEP, the intratidal gas distribution to the dependent region was higher than that to the non-dependent region. Decreasing the PEEP level resulted in a higher overall gas distribution to the non-dependent region compared with the dependent region. At a PEEP level of 10 cm H_2_O, the intratidal gas distribution curves of both regions crossed each other during a breath (Figure 4). Figure 5 shows the calculated ITV index as percentage of 1 for each PEEP level in each individual patient. There was a correlation between the PaO_2_/FiO_2_ ratio and the ITV index (−0.762; *P* <0.001).

## Discussion

This study demonstrates that intratidal gas distribution visualizes the best PEEP as compared with dynamic compliance in post cardiac surgery patients. In addition, the ITV index is able to determine a specific PEEP level in each individual patient, resulting in an even distribution of tidal volume to the non-dependent and dependent lung regions. Below this specific PEEP level, the intratidal gas distribution is predominantly distributed to the non-dependent region. This indicates that, at these PEEP levels, there is less ventilation in the dependent region due to lung collapse. In contrast, at PEEP levels above this specific level, there is less ventilation in the non-dependent region indicating overdistention.

In an experimental study, Protti *et al*. showed that ventilation with high tidal volumes, resulting in an expiratory volume of 1.5 times FRC (equal to a strain of 1.5), caused severe lung edema; all their study animals died within the observation period of 54 h [22]. In a second study, the authors ventilated all animals with a strain of 2.5 and showed that high tidal volumes with a low level of PEEP damaged the lungs and increased mortality, whereas high PEEP levels together with low tidal volume but with the same strain of 2.5, did not result in edema and all animals in this group survived [23]; it was suggested that application of high PEEP levels might lead to more homogeneous lung ventilation. In 1970, Mead *et al*. estimated that forces acting on lung tissue increase with a factor 4.5 when lungs are inhomogeneously ventilated [24]. This was recently confirmed by Rausch *et al*. who performed x-ray tomographic microscopy (generating detailed three-dimensional alveolar geometry) in rat lungs and found local strain values four times the global strain [25]. Therefore, a parameter that describes the ventilation distribution could be of importance in finding the best PEEP in patients with acute respiratory distress syndrome (ARDS).

Intratidal gas distribution was first described by Löwhagen *et al*. who used this technique in 16 volume-controlled ALI/ARDS patients to describe ventilation distribution to different lung regions within an inspiration [14]; they found that the intratidal gas distribution of the dorsal and mid-dorsal regions increased at higher PEEP levels, indicating redistribution of ventilation to the dependent region [14]. We modified their analysis by combining the ventral and mid-ventral regions into a non-dependent region and their mid-dorsal and dorsal regions into a dependent region [26]. Previously, we used the intratidal gas distribution technique to assess the effect of different assist levels during pressure support ventilation (PSV) and neurally adjusted ventilatory assist (NAVA) on ventilation distribution. We demonstrated that NAVA improved ventilation of the dependent lung region compared with PSV, leading to a more homogeneous ventilation of the lung [26]. In that study using the intratidal gas distribution technique, we demonstrated for the first time, less over-assistance during NAVA whereas there was marked over-assistance at higher pressure support levels [26]. This latter finding indicates that PSV with higher support levels mimics control ventilation with predominantly ventilation of the non-dependent lung. We also used the intratidal gas distribution in an experimental study comparing global and regional parameters to detect best PEEP in healthy and in ARDS lungs [27]. In these animals we found the same trend as in the present study, that is, that the intratidal gas distribution curves of the dependent and non-dependent regions reached each other at a specific PEEP level. Below this specific PEEP level, ventilation is mainly distributed to the non-dependent lung and above this level ventilation is mainly distributed to the dependent lung region. Above this specific PEEP level ventilation distribution to the non-dependent lung region diminished at higher PEEP levels, indicating that this region is overdistended.

In the present study we used a decremental PEEP trial and, as long as the PEEP level is adequate to keep the dependent lung region open, the used pressure amplitude of around 10 cm H_2_O was sufficient to ventilate this region; however, after collapse the inspiratory pressures are too low to open up this dependent region. This indicates that ventilation distribution to the dependent lung will be improved if higher inspiratory pressures are used.To make the intratidal gas distribution an easy-to-use parameter at the bedside, we introduced the ITV index. An ITV index of 1 indicates homogeneous distribution of tidal volume to the non-dependent and dependent lung regions. The intratidal gas distribution shows the behavior of the distribution of tidal volume during one breath. Figure 5 illustrates the ITV index, in which 100% indicates an ITV index of 1, that is, an equal distribution of ventilation to the dependent and non-dependent lung regions.

To test whether the intratidal gas distribution reliably defines the optimal PEEP, we compared the optimal PEEP values defined by the intratidal gas distribution, PaO_2_/FiO_2_ ratio and dynamic compliance. We found a negative correlation between the PaO_2_/FiO_2_ ratio and the ITV index. A lower ITV index means that more tidal ventilation is distributed to the better perfused dependent lung region, leading to less shunt and improved PaO_2_/FiO_2_ ratio. In contrast, we found no correlation between ITV and dynamic compliance. Alveoli that open up during inspiration but collapse during expiration give higher compliance values, whereas this cyclic collapse is harmful to the lung. In addition, recruitment of a lung region, but collapse in another lung region at the same time, will not increase dynamic compliance of the entire lung. This latter effect can be visualized by regional or pixel compliance from EIT. Costa *et al*. introduced the regional or pixel compliance based on EIT measurements during a decremental PEEP trial in two patients with pneumonia [12]. These patients were ventilated with PCV using a constant pressure amplitude and the change in impedance represents the change in volume; thus, for each pixel the compliance could be calculated. The highest compliance at a specific PEEP level was indicated as the best PEEP. Above this PEEP value, compliance decreased due to over-distention and below this value compliance decreased due to collapse. The authors showed that the optimal PEEP level was different for the dependent and non-dependent region [12]. Also, in their experimental study, Dargaville *et al*. demonstrated that ventral, medial and dorsal lung regions have different optimal PEEP levels based on regional compliance values in both normal and surfactant-depleted lungs [28]. This was confirmed by our results, in which the best PEEP level for the non-dependent and dependent regions were 5 and 15 cm H_2_O, respectively (Figure 2B).

In accordance with our previous studies [16,29], we found more ventilation of the dependent region at higher PEEP levels, as described by the parameter COV (Figure 2D). COV describes the ventilation distribution in the ventral-to-dorsal direction and a value of 50% reflects an even distribution [13]. Thus, COV provides information about the optimal distribution of tidal volume to the non-dependent and dependent regions at a certain PEEP level, but it does not give information about collapse or overdistention during a breath, as is seen with the intratidal gas distribution.

The RVD was developed to assess the homogeneity of aeration of the lung regions [15,19] during a slow inflation maneuver. It has been demonstrated in pigs that ALI lungs are more inhomogeneous compared with healthy lungs [21]; however, application of higher PEEP levels improved the homogeneity of lung ventilation. In addition, the authors showed that the dependent region was slower inflated as compared with the non-dependent region, indicating inhomogeneous ventilation of the dorsal lung parts; however, they used the SDs of the RVD index to create a ventilation homogeneity map [21]. In the present study we calculated the time needed to reach a threshold of 40% of the regional impedance change compared with the total inspiratory time and without the use of a slow flow inflation. We found that RVD values in both lung regions increased at each decremental PEEP step. Therefore, we were unable to detect the best PEEP level by means of RVD, with the used PEEP levels. However, Wrigge *et al*. described that RVD could only be used during a slow flow inflation maneuver with a tidal volume of 12 mL/kg to describe tidal recruitment [15]. Therefore, in the present study the results of the RVD are incorrect. Another index to describe homogeneous ventilation is the GI index, which quantifies the variation in tidal ventilation distribution [20,21] and shows the lowest value in healthy patients and the highest value in patients with ARDS [21]. However, because this index describes homogeneity based on differences in measured impedance, the index value does not take into account the presence of atelectasis or over-distention and, therefore, we believe that this is not an appropriate index. As within our range of PEEP levels we were unable to detect an optimal PEEP level based on the GI index and RVD index, it is possible that an optimum may have been found if higher PEEP levels had been applied than used in the present study.

This study has some limitations. First, EIT measures ventilation distribution in a lung slice of approximately 5 to 10 cm [30,31]; therefore, information gathered by EIT has a limited external validity for the remaining lung tissue. However, placing the EIT belt at a higher position is known to reduce the probability to detect inhomogeneity of the lungs at decreasing PEEP levels [16] and, thus, the probability to detect lung areas susceptible to the development of VILI. Therefore, we placed the EIT belt just above the diaphragm to increase the probability of detecting lung collapse.

Second, based on the protocol of our study described previously, EIT measurements were not recorded continuously [16]. However, by treating the EIT data as percentages (instead of absolute values) the baseline shifts are corrected. In addition, the EIT belt was not disconnected from the patient to measure the same lung slice during each measurement. Third, this study was performed with post cardiac surgery patients who respond well to a recruitment maneuver. However, Reis Miranda *et al*. demonstrated that a PEEP level of 15 cm H_2_O was necessary to keep the lung open (PaO_2_/FiO_2_ > 350 mmHg) in cardiac surgery patients [32]; the authors also showed that if patients are ventilated according to the open lung concept, fewer cytokines are released, FRC recovers faster, less oxygen is required in the normal ward, and that afterload of the right ventricle is lower in these patients [32-35]. Despite the fact that adequate PEEP settings are also important in cardiac surgery patients, most patients in need of a recruitment maneuver in combination with the best PEEP are ARDS patients. However, ARDS patients show less response to a recruitment maneuver compared with cardiac surgery patients, due to fewer gravitational-dependent infiltrates. Particularly in ARDS patients it is important to achieve homogeneous ventilation to reduce the stress acting on lung tissue. Our earlier experimental study showed the same trend of tidal ventilation distribution during a decremental PEEP trial in both ARDS-induced and healthy lungs [27]. However, additional studies are required to test whether application of this specific PEEP level leads to better outcome and also to confirm the present findings in patients with ARDS.

## Conclusion

The ITV index was comparable with dynamic compliance to indicate the best PEEP level in post cardiac surgery patients. The intratidal gas distribution is able to identify the onset of over-distention in the non-dependent part and recruitment in the dependent part. We believe that the ITV index may be the ideal bedside tool to detect the best PEEP; however, its preventive effect on ventilator-induced lung injury (VILI) and thereby on outcome still needs to be examined in patients with ALI/ARDS.

## Key messages

● Several EIT parameters have recently been developed to optimize ventilator settings.

● Tidal impedance variation, regional compliance and ventilation surface area showed different optimal PEEP levels for the dependent and non-dependent lung regions.

● The intratidal gas distribution is able to detect the onset of alveolar overdistention and collapse, within one inspiration.

● Best PEEP as defined by the intratidal gas distribution shows good agreement with best PEEP as defined by the global parameter dynamic compliance.

● Equal distribution of ventilation to the dependent and non-dependent lung regions, as defined by the intratidal gas distribution, might lower stress and strain in the lung.

## Abbreviations

ALI: acute lung injury; ANOVA: analysis of variance; ARDS: acute respiratory distress syndrome; COV: center of ventilation; CT: computed tomography; EIT: electrical impedance tomography; FiO_2_: inspired oxygen fraction; FRC: functional residual capacity; GI index: global inhomogeneity index; ITV index: intratidal gas distribution index; NAVA: neurally adjusted ventilatory assist; PaO_2_: partial arterial oxygen pressure; PCV: pressure-controlled ventilation; PEEP: positive end-expiratory pressure; PIP: peak inspiratory pressure; PSV: pressure support ventilation; RVD index: regional ventilation delay index; TIV: tidal impedance variation; VILI: ventilator-induced lung injury; VSA: ventilation surface area.

## Competing interests

PB: the author declares that he has no competing interests. DH: the author declares that he has no competing interests. EGJ: the author declares that he has no competing interests. DG: received fees from Draeger for oral presentations.

## Authors’ contributions

PB: carried out the data acquisition, data processing, data analysis, and statistical analysis and participated in drafting the manuscript. DH: carried out statistical analysis and participated in drafting the manuscript. EGJ: carried out the data analysis and participated in drafting the manuscript. DG: participated in data acquisition and drafting the manuscript. All authors read and approved the final version of the manuscript.

## References

[B1] Gattinoni L Carlesso E Caironi P Stress and strain within the lung Curr Opin Crit Care 2012 18 42 47 10.1097/MCC.0b013e32834f17d9 22157254

[B2] Suter PM Fairley B Isenberg MD Optimum end-expiratory airway pressure in patients with acute pulmonary failure N Engl J Med 1975 292 284 289 10.1056/NEJM197502062920604 234174

[B3] Tusman G Suarez-Sipmann F Bohm SH Pesch T Reissmann H Meschino G Scandurra A Hedenstierna G Monitoring dead space during recruitment and PEEP titration in an experimental model Intensive Care Med 2006 32 1863 1871 10.1007/s00134-006-0371-7 17047925

[B4] Suarez-Sipmann F Bohm SH Tusman G Pesch T Thamm O Reissmann H Reske A Magnusson A Hedenstierna G Use of dynamic compliance for open lung positive end-expiratory pressure titration in an experimental study Crit Care Med 2007 35 214 221 10.1097/01.CCM.0000251131.40301.E2 17110872

[B5] Carvalho AR Jandre FC Pino AV Bozza FA Salluh J Rodriques R Ascoli FO Giannella-Neto A Positive end-expiratory pressure at minimal respiratory elastance represents the best compromise between mechanical stress and lung aeration in oleic acid induced lung injury Crit Care 2007 11 R86 10.1186/cc6093 17688701 PMC2206498

[B6] Maisch S Reissmann H Fuellekrug B Weismann D Rutkowski T Tusman G Bohm SH Compliance and dead space fraction indicate an optimal level of positive end-expiratory pressure after recruitment in anesthetized patients Anesth Analg 2008 106 175 181 10.1213/01.ane.0000287684.74505.49 18165575

[B7] Henzler D Mahnken AH Wildberger JE Rossaint R Gunther RW Kuhlen R Multislice spiral computed tomography to determine the effects of a recruitment maneuver in experimental lung injury Eur Radiol 2006 16 1351 1359 10.1007/s00330-005-0003-6 16220208

[B8] Pelosi P Rocco PR de Abreu MG Use of computed tomography scanning to guide lung recruitment and adjust positive-end expiratory pressure Curr Opin Crit Care 2011 17 268 274 10.1097/MCC.0b013e328344ddbc 21415738

[B9] Frerichs I Hinz J Herrmann P Weisser G Hahn G Dudykevych T Quintel M Hellige G Detection of local lung air content by electrical impedance tomography compared with electron beam CT J Appl Physiol 2002 93 660 666 12133877 10.1152/japplphysiol.00081.2002

[B10] Meier T Luepschen H Karsten J Leibecke T Grossherr M Gehring H Leonhardt S Assessment of regional lung recruitment and derecruitment during a PEEP trial based on electrical impedance tomography Intensive Care Med 2008 34 543 550 10.1007/s00134-007-0786-9 17653529

[B11] Victorino JA Borges JB Okamoto VN Matos GF Tucci MR Caramez MP Tanaka H Sipmann FS Santos DC Barbas CS Carvalho CR Amato MB Imbalances in regional lung ventilation: a validation study on electrical impedance tomography Am J Respir Crit Care Med 2004 169 791 800 10.1164/rccm.200301-133OC 14693669

[B12] Costa EL Borges JB Melo A Suarez-Sipmann F Toufen C Jr Bohm SH Amato MB Bedside estimation of recruitable alveolar collapse and hyperdistension by electrical impedance tomography Intensive Care Med 2009 35 1132 1137 10.1007/s00134-009-1447-y 19255741

[B13] Frerichs I Dargaville PA van Genderingen H Morel DR Rimensberger PC Lung volume recruitment after surfactant administration modifies spatial distribution of ventilation Am J Respir Crit Care Med 2006 174 772 779 10.1164/rccm.200512-1942OC 16840739

[B14] Lowhagen K Lundin S Stenqvist O Regional intratidal gas distribution in acute lung injury and acute respiratory distress syndrome assessed by electric impedance tomography Minerva Anestesiol 2010 76 1024 1035 21178912

[B15] Wrigge H Zinserling J Muders T Varelmann D Gunther U von der Groeben C Magnusson A Hedenstierna G Putensen C Electrical impedance tomography compared with thoracic computed tomography during a slow inflation maneuver in experimental models of lung injury Crit Care Med 2008 36 903 909 10.1097/CCM.0B013E3181652EDD 18431279

[B16] Bikker IG Preis C Egal M Bakker J Gommers D Electrical impedance tomography measured at two thoracic levels can visualize the ventilation distribution changes at the bedside during a decremental positive end-expiratory lung pressure trial Crit Care 2011 15 R193 10.1186/cc10354 21834953 PMC3387635

[B17] Adler A Amyot R Guardo R Bates JH Berthiaume Y Monitoring changes in lung air and liquid volumes with electrical impedance tomography J Appl Physiol 1997 83 1762 1767 9375349 10.1152/jappl.1997.83.5.1762

[B18] Frerichs I Hahn G Schiffmann H Berger C Hellige G Monitoring regional lung ventilation by functional electrical impedance tomography during assisted ventilation Ann NY Acad Sci 1999 873 493 505 10.1111/j.1749-6632.1999.tb09498.x 10372185

[B19] Muders T Luepschen H Zinserling J Greschus S Fimmers R Guenther U Buchwald M Grigutsch D Leonhardt S Putensen C Wrigge H Tidal recruitment assessed by electrical impedance tomography and computed tomography in a porcine model of lung injury Crit Care Med 2012 40 903 911 10.1097/CCM.0b013e318236f452 22202705

[B20] Zhao Z Moller K Steinmann D Frerichs I Guttmann J Evaluation of an electrical impedance tomography-based Global Inhomogeneity Index for pulmonary ventilation distribution Intensive Care Med 2009 35 1900 1906 10.1007/s00134-009-1589-y 19652949

[B21] Zhao Z Steinmann D Frerichs I Guttmann J Moller K PEEP titration guided by ventilation homogeneity: a feasibility study using electrical impedance tomography Crit Care 2010 14 R8 10.1186/cc8860 20113520 PMC2875520

[B22] Protti A Cressoni M Santini A Langer T Mietto C Febres D Chierichetti M Coppola S Conte G Gatto S Leopardi O Masson S Lombardi L Lazzerini M Rampoldi E Cadringher P Gattinoni L Lung stress and strain during mechanical ventilation: any safe threshold? Am J Respir Crit Care Med 2011 183 1354 1362 10.1164/rccm.201010-1757OC 21297069

[B23] Protti A Andreis DT Monti M Santini A Sparacino CC Langer T Votta E Gatti S Lombardi L Leopardi O Masson S Cressoni M Gattinoni L Lung stress and strain during mechanical ventilation: any difference between statics and dynamics? Crit Care Med 2013 41 1046 1055 10.1097/CCM.0b013e31827417a6 23385096

[B24] Mead J Takishima T Leith D Stress distribution in lungs: a model of pulmonary elasticity J Appl Physiol 1970 28 596 608 5442255 10.1152/jappl.1970.28.5.596

[B25] Rausch SM Haberthur D Stampanoni M Schittny JC Wall WA Local strain distribution in real three-dimensional alveolar geometries Ann Biomed Eng 2011 39 2835 2843 10.1007/s10439-011-0328-z 21607757

[B26] Blankman P Hasan D van Mourik MS Gommers D Ventilation distribution measured with EIT at varying levels of pressure support and Neurally Adjusted Ventilatory Assist in patients with ALI Intensive Care Med 2013 39 1057 1062 10.1007/s00134-013-2898-8 23553568

[B27] Bikker IG Blankman P Specht P Bakker J Gommers D Global and regional parameters to visualize the ‘best’ PEEP during a PEEP trial in a porcine model with and without acute lung injury Minerva Anestesiol 2013 79 983 992 23811623

[B28] Dargaville PA Rimensberger PC Frerichs I Regional tidal ventilation and compliance during a stepwise vital capacity manoeuvre Intensive Care Med 2010 36 1953 1961 10.1007/s00134-010-1995-1 20689920

[B29] Bikker IG Leonhardt S Bakker J Gommers D Lung volume calculated from electrical impedance tomography in ICU patients at different PEEP levels Intensive Care Med 2009 35 1362 1367 10.1007/s00134-009-1512-6 19513694 PMC2712617

[B30] Erlandsson K Odenstedt H Lundin S Stenqvist O Positive end-expiratory pressure optimization using electric impedance tomography in morbidly obese patients during laparoscopic gastric bypass surgery Acta Anaesthesiol Scand 2006 50 833 839 10.1111/j.1399-6576.2006.01079.x 16879466

[B31] Lindgren S Odenstedt H Olegard C Sondergaard S Lundin S Stenqvist O Regional lung derecruitment after endotracheal suction during volume- or pressure-controlled ventilation: a study using electric impedance tomography Intensive Care Med 2007 33 172 180 10.1007/s00134-006-0425-x 17072587

[B32] Reis MD Struijs A Koetsier P van Thiel R Schepp R Hop W Klein J Lachmann B Bogers AJ Gommers D Open lung ventilation improves functional residual capacity after extubation in cardiac surgery Crit Care Med 2005 33 2253 2258 10.1097/01.CCM.0000181674.71237.3B 16215379

[B33] Reis MD Gommers D Struijs A Meeder H Schepp R Hop W Bogers A Klein J Lachmann B The open lung concept: effects on right ventricular afterload after cardiac surgery Br J Anaesth 2004 93 327 332 10.1093/bja/aeh209 15247107

[B34] Reis MD Gommers D Struijs A Dekker R Mekel J Feelders R Lachmann B Bogers AJ Ventilation according to the open lung concept attenuates pulmonary inflammatory response in cardiac surgery Eur J Cardiothorac Surg 2005 28 889 895 10.1016/j.ejcts.2005.10.007 16271479

[B35] Reis MD Klompe L Mekel J Struijs A van Bommel J Lachmann B Bogers AJ Gommers D Open lung ventilation does not increase right ventricular outflow impedance: An echo-Doppler study Crit Care Med 2006 34 2555 2560 10.1097/01.CCM.0000239118.05093.EE 16932227

